# Psychosocial working conditions and the risk of depression and anxiety disorders in the Danish workforce

**DOI:** 10.1186/1471-2458-8-280

**Published:** 2008-08-07

**Authors:** Joanna Wieclaw, Esben Agerbo, Preben Bo Mortensen, Hermann Burr, Finn Tuchsen, Jens Peter Bonde

**Affiliations:** 1 Department of Occupational Medicine, Aarhus University Hospital, Aarhus, Norrebrogade 44, bygning 2C, DK 8000 Aarhus C, Denmark; 2 National Centre for Register-Based Research, Aarhus University, Aarhus, Taasingegade 1, DK 8000 Aarhus C, Denmark; 3 National research Centre for the Working Environment, Lersø Parkallé 105, DK 2100 København Ø, Denmark

## Abstract

**Background:**

To examine the risk of depressive and anxiety disorders according to psychosocial working conditions in a large population-based sample.

**Methods:**

Job Exposure Matrix was applied to assess psychosocial working conditions in a population-based nested case-control study of 14,166 psychiatric patients, diagnosed with depressive or anxiety disorders during 1995–1998 selected from The Danish Psychiatric Central Research Register, compared with 58,060 controls drawn from Statistics Denmark's Integrated Database for Labour Market Research.

**Results:**

Low job control was associated with an increased risk of anxiety disorders in men (IRR 1.40, 95% CI 1.24–1.58).

In women an elevated risk of depression was related to high emotional demands (IRR 1.39, 95%CI 1.22–1.58) and to working with people (IRR 1.15, 95% CI 1.01–1.30). In both sexes high demands were associated with a decreased risk of anxiety disorders. There was a weak association between job strain and anxiety disorders in men (IRR 1.13, 95%, CI 1.02–1.25)

**Conclusion:**

Psychosocial work exposures related to the risk of depressive and anxiety disorders differ as between the sexes. The pattern of risks is inconsistent. The results give rise to rethinking both study designs and possible causal links between work exposures and mental health.

## Background

Studies of psychosocial work environment based on the control-demand-support model [1,2] indicate that lack of job control, low decision authority, low skill discretion and job strain (a combination of high demands and low control) are associated with the risk of depression, anxiety, distress, fatigue, job dissatisfaction, burn-out and sickness absence [3-7].

Other work-related factors that have been shown to be associated with psychological distress and depression, especially among human service professionals, are emotional demands [8-10], work conflicts, job insecurity [11,12], managerial styles, organisational justice and climate [13,14] as well as exposure to threats and violence [15-18].

Most of these studies are cross-sectional and confined to a selected occupation or sector, rendering causal interpretations and generalisation difficult.

Longitudinal studies provide some support for effects of job demands, control, job strain and to a lesser extent social support on mental health outcomes [5,19-21].

Epidemiological studies of health risk related to the psychosocial work environment are facing challenges regarding reliable exposure assessment. Limitations of widely used self-reports include common method variance and recall bias in studies with retrospective exposure data collection [22]. These limitations may severely undermine the validity of findings and render causal inferences difficult [23].

Job Exposure Matrices (JEM), where occupational titles are used to assign the types and levels of occupational exposures, reduce the measurement variation and bypass the problem of recall bias because the exposure is assigned independently of the case status [24,25].

JEMs can easily be used in large population studies. Most JEMs concern physical and chemical exposures; however a few also include ergonomic and psychosocial risk factors [24,26-28].

A limited number of psychosocial work measures have been found to be associated with occupation, and thus suitable for JEM. Decision authority, skill discretion and job control and to lesser degree emotional demands have been shown to have a high variation across occupations whereas job demands, social support and job insecurity, are less discriminative of occupation [26,29,30].

Denmark has a long tradition of collecting data on both somatic and mental health outcomes through population based registers (National Patient Register and Central Psychiatric Register). However, data on occupational exposures are usually not directly available or are difficult and costly to obtain, especially in large epidemiological studies. Often, the only information on exposure is the occupational title or the industrial sector in which a person is employed. In such a case the JEM may be an appropriate study method.

Few health outcomes have been studied using psychosocial JEM. Both a Swedish and a Danish study found that job strain, low job control and low skill discretion are associated with an increased risk of cardiovascular mortality and myocardial infraction [31-33]. Low control, low social support at work along with passive job environment (low demands and low control) were related to a risk of alcoholism in Swedish men [34]. Low job control predicted all causes mortality in an American study [28] and a German study that applied the Finish JEM (FINJEM) showed that having a challenging job and job control had the protective effect on the development of dementia [35].

We have previously shown that the risk of depressive and anxiety disorders varies across occupations [36] and we believe that differences in psychosocial working conditions may provide some explanation for these findings. The present nested case-control study examines the relationship between selected psychosocial work conditions assessed by a Job Exposure Matrix and the risk of psychiatrically diagnosed depressive and anxiety disorders in the Danish working population. These exposures have not, to our knowledge, been previously examined by means of JEM, in a large epidemiological study.

## Methods

### Study sample

Cases for this population-based nested case-control study were selected among all patients recorded in the Danish Psychiatric Central Research Register, aged 18–65, who in the period 1 January 1995 to 31 December 1998 have received a first-ever diagnosis of an depressive or anxiety disorder.

Five never-admitted referents of the same gender and age were selected for each case, using the incidence density risk set matching method [37], in the Statistics Denmark's Integrated Database for Labour Market Research (IDA) 5% sample of the Danish population. The unique person identifier (Central Person Register number – CPR), which can be logically checked for errors, was used to identify and merge data across the registers.

The present study included only persons that had a job title and were currently registered as employed.

The study is a part of a larger project "Unemployment, occupation and mental disorders", conducted at the National Centre for Register-based Research, which has received the approval of The Danish Ethical Committee. Anonymised register data used in the present study can be requested for scientific purposes by contacting the authors.

### Outcome measure

Our outcome measure was the first-ever clinical diagnosis, according to Word Health Organisation International Classification of Diseases version 10 (WHO ICD-10), of affective disorder (F30–39) or anxiety disorder (F40–48) made by a psychiatrist in charge of hospital or outpatient treatment. Both diagnostic categories consist of several sub-diagnoses: F 30–39 mainly different forms of depressive conditions: bipolar affective disorders, depressive episode and recurrent depression while F 40–48 includes various anxiety disorders: phobic anxiety disorder, other anxiety disorders, reaction to severe stress, adjustment disorders and somatoform disorders. We decided to use the broad diagnostic categories, as they are believed to be more reliable than the more specific sub-categories [38]. As there are no private psychiatric hospitals in Denmark we had a complete record of all cases in our study period.

### Job classification

The occupation held the year before the matching date was the exposure measure. Occupational codes were extracted for all subjects according to the Danish version of the International Standard Classification of Occupations (DISCO 88) from the IDA database. This classification system is based on skills/education required and the actual job task performed. Occupations are divided into four hierarchical levels, where each additional digit in the DISCO code indicates a more specific job category. Employers are obliged to submit employees' DISCO occupational codes to the National Salary Register, where they are subsequently validated against several other registers in Statistics Denmark.

The time of cases being diagnosed was the anchor point for the job held by both cases and referents.

Occupation was used as a proxy for exposure to psychosocial work conditions contained in the JEM constructed for the purpose of the present study.

### Job Exposure Matrix

The JEM was constructed from data in the Danish Work Environment Cohort Study (DWECS) carried by the National Institute of Occupational Health. Data regarding different aspects of physical and psychosocial work environment have, since 1990, been collected every fifth year by a telephone interview of a random representative sample of the Danish population [39]. In the present study we used cross-sectional data on 5 387 employees, aged 18–69, who had complete occupational and demographical records in year 2000. The latest DWECS was chosen because it contains better measures of psychosocial work variables than earlier additions and because we have data showing that these variables have been stable over our study period [39].

The psychosocial work variables selected from DWECS were found in the international literature to be possible predictors of depression and anxiety [5,19,40,41,21]. They included dimensions of the demand-control model as well as exposure to emotional demands and working with people (clients, customers, students, pupils). Data regarding the demand-control model were collected on the basis of an adopted version of the Job Content Questionnaire [1] containing five 3–5 questions scales and single item questions with 4–6 response options.

Emotional demands were assessed with 3 questions "Does your work put you in emotionally disturbing situations?, "Is your work emotionally demanding?", "Do you get emotionally involved in your work?" with answer possibilities "Always", "Often", "Seldom", "Never/hardly ever", while working with people was measured with a single question "Do you deal with people (clients, customers, students, pupils) who are not employed at your workplace when carrying out your work?" with answer possibilities "Almost all working hours", "3/4 working hours", "1/2 working hours", "1/4 working hours", "Seldom". "Never".

Reliability of the scales as measured by Chronbachs alpha was above 60%. Responses were scored with equal weight and equal intervals between options and then transformed to a 0–100 rating scale (for more detailed description of the scales se Rugulies et al [42] and Kristensen et al. [43]). Job control scores were calculated as a mean of decision authority and skill discretion scores.

On the basis of individual scores, the mean was calculated for each DISCO occupational group (on the 2–4-digit DISCO code level).

To obtain scores on job strain, which is conceptualised in the model as a combination of exposure to high job demands and low job control, we have calculated for each job category the proportion of persons that have reported both high job demands – score above the highest tertile on the demands scale, and low job control – score below the lowest tertile on the control scale.

The final gender stratified JEM included 5 variables representing the control-demand model (decision authority, skills discretion, job demands, job control and job strain) and variables emotional demands and working with people (clients, customers, students, pupils).

Subsequently, we merged the JEM data with our study sample by occupation and gender, so that each person was assigned the mean value of the JEM exposure on the basis of his/her occupational title. The final JEM included 85 and 82 occupational categories for women and men respectively, with a minimum of 10 observations in a group. In the analyses we used exposure data at the most detailed DISCO code available to be able to analyse as narrow as possible job categories.

Exposure data were then categorised into 4 exposure levels by quartiles based on the distribution of scores among the controls. In the case of decision authority, skill discretion and job control high exposure level was used as a reference, while low exposure was reference group for job demands, emotional demands and working with people. Strain variable was dichotomised; jobs with the prevalence of strain higher than 20% were defined as high strain jobs.

### Statistical analysis

The incidence rate ratios (IRR) of depressive and anxiety disorders were calculated for each of the psychosocial variables, using the conditional logistic regression model for nested case-control data [37,44] (PhReg procedure, the SAS version 8). In a nested case-control study IRR can be interpreted as relative risk (RR) [44], thus this term will be used in the paper (shorthand: risk). The IRR were adjusted for gender, age and calendar time by stratification and socio-demographic covariates: marital status (single/not single), having children living at home (yes/no), socio-economic-status (level of education and annual income), total duration of unemployment (less/more than 2 years), citizenship (Danish/not Danish) and place of residence (urban/rural and a geographical location) by regression. All covariates were included at the same time and kept in the model for all analyses.

## Results

The study included 14.166 cases and 58.060 controls; sixty two percent were women. 67% of cases were diagnosed with anxiety disorders whereas 33% were diagnosed with depressive disorders. The high proportion of anxiety disorders reflects the fact that cases include both in and out patients with first ever psychiatric diagnosis. Age span was 18–65, and depression was more frequent among cases older than 40 years (53%) while proportion of anxiety disorders was higher among cases younger than 40 years (66%). Being single, having low income and living outside a bigger town was associated with the risk of both disorders in both sexes (data not shown).

**Figure 1:**
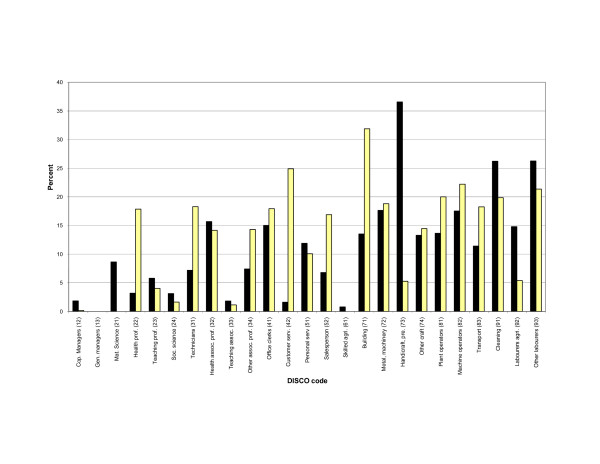
**Prevalence of job strain according to DISCO 2-digit code occupational groups**. Yellow bars: Women. Black bars: Men.

All variables showed a considerable variation across occupations (data not shown). Skill discretion had the highest mean scores and the lowest variation across DISCO 2-digit occupational groups, whereas working with people and emotional demands had the largest variation. Decision authority and job control had parallel patterns and showed a declining tendency with more industrial and less skilled jobs. Job strain varied considerably across occupations as shown in figure 1.

The risks related to specific psychosocial variables are presented in tables 1 and 2.

**Table 1:** Adjusted* incidence rate ratios of depressive and anxiety disorders according to exposure to psychosocial risk factors at work. Women

**Prevalence %**	**Depressive disorders**				**Anxiety disorders**			
		
**Psychosocial risk factor**	**Controls**	**Cases**	**IRR adj.**	**CI 95%**	**Controls**	**Cases**	**IRR adj.**	**CI 95%**
**Job control**								
low <= 54	2640	699	0.95	0.83–1.10	5419	1518	1.01	0.91–1.11
54 < medium <= 62.5	3176	733	0.93	0.82–1.07	5983	1410	0.89	0.81–0.98
62.5 < medium-high <= 67	2866	833	**1.15**	**1.02–1.30**	5708	1489	0.99	0.90–1.09
high > 67 (ref)	3004	742	1		5483	1327	1	
**Job demands**								
high > 39.3	3076	664	0.89	0.78–1.02	5867	1226	**0.84**	**0.77–0.93**
39.3 >= medium-high > 34.3	2953	660	**0.87**	**0.77–0.99**	5713	1339	**0.86**	**0.79–0.95**
34.3 >= medium > 31	2915	924	**1.20**	**1.07–1.35**	5613	1674	1.09	1.00–1.19
low <= 31 (ref)	2742	759	1		5400	1505	1	
**Job Strain**								
Yes > 20	2677	726	1.01	0.92–1.12	5009	1367	1.04	0.97–1.12
No <= 20	9009	2281	1		17584	4377	1	
**Emotional demands**								
high > 47.3	2980	904	**1.39**	**1.22–1.58**	5440	1374	1.07	0.97–1.18
47.3 >= medium-high > 29.6	2820	740	1.13	0.99–1.28	5449	1449	1.03	0.94–1.12
29.6 >= medium > 21.5	3010	630	0.94	0.83–1.07	5925	1380	0.88	0.80–0.96
low <= 21.5 (ref)	2876	733	1		5779	1541	1	
**Working with people**								
high > 73.4	3113	871	**1.15**	**1.01–1.30**	5885	1426	1.03	0.94–1.13
61.8 < medium-high <= 73.4	2557	695	1.13	0.99–1.28	4937	1358	1.07	0.98–1.17
38.2 < medium <= 61.8	3357	768	0.99	0.88–1.12	6417	1615	1.01	0.93–1.10
low <= 38.2 (ref)	2659	673	1		5354	1345	1	

* IRRs are adjusted for marital status (single yes/no), having children (yes/no), level of education (up to vocational/higher), income level (low/high), total level of unemployment (less than 2 years/over 2 years) residence (town/province) and nationality (Danish/not Danish) **Bold **types indicate significant IRRs

**Table 2:** Adjusted* incidence rate ratios of depressive and anxiety disorders according to exposure to psychosocial risk factors at work. Men

**Prevalence %**	**Depressive disorders**				**Anxiety disorders**			
		
**Psychosocial risk factor**	**Controls**	**Cases**	**IRR adj.**	**CI 95%**	**Controls l**	**Cases**	**IRR adj.**	**CI 95%**
**Job control**								
low <= 56	1962	505	1.05	0.90–1.21	3670	1033	**1.40**	**1.24–1.58**
56 < medium <= 65.5	2072	483	0.98	0.84–1.13	3969	917	1.13	1.00–1.27
65.5 < medium-high <= 72.8	2275	496	0.93	0.80–1.07	3718	751	1.11	0.99–1.26
high > 72.8 (ref)	2455	557	1		3660	673	1	
**Job demands**								
high > 75	2329	507	0.88	0.76–1.02	3706	717	**0.79**	**0.70–0.89**
38.4 < medium-high <= 75	2379	511	**0.86**	**0.74–0.99**	4064	795	**0.81**	**0.72–0.90**
35.8 < medium <= 38.4	1881	445	0.92	0.79–1.06	3323	790	0.93	0.84–1.04
low <= 35.8 (ref)	2175	578	1		3924	1072	1	
**Job Strain**								
yes > 20	1381	341	1.01	0.88–1.17	2546	682	**1.13**	**1.02–1.25**
No <= 20	7383	1700	1		12471	2692	1	
**Emotional demands**								
high > 28.6	2350	604	1.12	0.96–1.30	3649	883	1.12	1.00–1.26
18.6 < medium-high <= 28.6	2129	413	0.93	0.80–1.09	3438	724	0.95	0.84–1.07
13.8 < medium <= 18.6	2217	515	1.02	0.89–1.18	3960	844	0.91	0.82–1.02
low <= 13.8 (ref)	2068	491	1		3970	923	1	
**Working with people**								
high > 63.5	2174	569	0.97	0.84–1.12	3595	933	1.10	098–1.23
40 < medium-high < 63.5	2374	488	**0.85**	**0.73–0.98**	3817	774	0.90	0.80–1.02
22 < medium <= 40	2163	441	**0.79**	**0.68–0.92**	3751	767	0.91	0.81–1.02
low <= 22 (ref)	2053	543	1		3854	900	1	

* IRRs are adjusted for marital status (single yes/no), having children (yes/no), level of education (up to vocational/higher), income level (low/high), total level of unemployment (less than 2 years/over 2 years) residence (town/province) and nationality (Danish/not Danish) **Bold **types indicate significant IRRs

Women working in jobs characterised by high emotional demands and in jobs where more than 63.5% of the time is spent working with people, had a significantly elevated risk of affective disorders. A significantly decreased risk of anxiety disorders was related to high and medium high job demands whereas the pattern for depression was inconsistent. A medium high level of job control was associated with an increase in the risk of depression.

In men low job control was associated with a significantly elevated risk of anxiety disorders. We found a weak association between risk of anxiety disorders and job strain. High and medium-high levels of job demands were associated with a significantly decreased risk of anxiety disorders, whereas significantly decreased risk of depression was related to medium-high and medium levels of working with people.

The association with the components of job control, decision authority and skill discretion, showed no clear pattern (data not shown). In women medium-high level of decision authority was associated with an increased risk of anxiety disorders (IRR 1.22, 95% CI 1.10–1.35) while in men low skill discretion was associated with an elevated risk of anxiety disorders (IRR 1.32, 95% CI 1.17–1.49)

## Discussion

We have examined the association between seven psychosocial work environment exposures and the risk of depressive and stress-related disorders in both sexes.

It is important to bear in mind that our findings apply to the risk of severe mental disorders that require hospital treatment and may not apply to milder cases of these disorders in the general population.

### The demand-control model

Men working in occupations with low job control had an elevated risk of anxiety disorders. This finding is in line with results of several other studies [6,11,12,45].

Contrarily to the prediction of the control-demand model, high job demands were in our study associated with the significantly decreased risk of anxiety disorders in both sexes, whereas the risk of depressive disorders showed a tendency to increase with lower levels of job demands. This result is unexpected and opposite to the findings of the Whitehall study [6] and the Gazel Cohort studies [45,46] in which high job demands were the strongest predictors of psychiatric disorders, especially for men. Equally, in a recent study by Melchior et al. psychological demands were associated with increased risks of depression and generalised anxiety in both sexes [47].

We have also found only the weak association between the risk of depression in men, but not in women, and job strain. This finding is not quite in line with the recent reviews, which concluded that job strain seem to be the strongest and the most consistent predictor of mental disorders [21,20]. We have no plausible explanation for these divergences. However, most of the reviewed studies concern common mental disorders and the discrepancy with our results may primarily reflect possible differences in the determinants of hospitalisation and milder disorders. Equally, the different pattern of risks in the sexes cannot be explained by data indicating different exposure patterns in men and women or by higher susceptibility for affective disorders in men. Therefore the elevated risk in one gender only, may be considered as an unexplained inconsistency in the results, which must be interpreted with caution. However, in an earlier study [48] we have shown that the risk of developing depressive and anxiety disorders was elevated in human service professions, and especially so among men employed in these professions. It is possible that the control-demand model, which mainly addresses quantitative and conflicting demands, may not quite capture qualitative and relational demands characteristic for these professions. Indeed, in our study, high levels of job strain can be seen in labour intensive occupations (se fig. 1).

The healthy worker effect may also influence the results, as person unable to cope with job demands could have been selected out of work, prior 1-year time lag used in our study.

### Emotional demands and working with people

The effect of "working with people" was different in the sexes; high exposure (more then 64% of the working hours) was associated with an increased risk of depression in women, while men with medium-high exposures had decreased risks of both disorders. In both sexes the risks showed a declining tendency with decreasing exposure. The result probably reflects the fact that women are more often employed in professions providing services to other people, which were previously found to carry an increased risk of both disorders [48].

High emotional demands were in women associated with an increased risk of depression. This result is in line with studies on "emotional labour" which suggest that dealing with emotional demands and a need to hide one's true emotions are risk factors for mental health problems, especially in human service occupations [49-51]. Surprisingly, emotional demands were not associated with the risk of anxiety disorders. We have no theoretically founded explanation for this finding and thus the results should be interpreted with caution. It can be hypothesized that anxiety disorders are more related to coping with external tasks and problems (traumatic exposures, stressful work conditions) whereas affective disorders may be related to more personal and interpersonal issues such as feelings of worthlessness and meaningfulness. As emotional demands concern professional responsibility for the welfare of other people, they may represent a greater risk regarding depression than anxiety disorders.

### Methodological issues

The strength of the present study is the nature of the outcome measure: the complete case record of clinically diagnosed mental disorders collected independently of the study, and the use of an independent, objective exposure measure. This approach ensures that the findings are not influenced by the fact that workers with mental health problems tend to perceive their work environment more negatively.

However, our findings are limited to severe, clinically diagnosed disorders and cannot be generalised to common mental health problems.

It is possible that individuals who suffer from difficult work conditions are more frequently hospitalised if they suffer from mental health problems, however we had no possibility to address this potential problem in our data analyses. If real, this phenomenon will tend to produce false positive results

Our results may also be influenced by problems related to the use of JEM.

Many psychosocial work environment variables are probably related more to individual work styles, preferences and habits as well as to work organisation than to an actual job function, and as such may be less suitable as work-related exposures [24,52]. Generally, variability of psychosocial exposures across occupations is rather low [24] and even though in our study, occupation explained a substantial proportion of variation in most variables (around 20%), the exposure contrast may still have been insufficient.

By assigning a mean value of exposure to all those employed in a particular occupation, we may have introduced some non-differential misclassification, as heterogeneity in psychosocial exposures within occupations is rather large owing to variation in actual work tasks and between worker variability [24,25]. Misclassification may have occurred despite the fact that about 70% of our exposure data were based on the most specific and homogenous DISCO 4-diget code occupational categories.

Assigning occupational scores to individuals minimises differential misclassification but ignores the fact that susceptibility to psychiatric disorders is related to a worker perception of, reaction to and interaction with working conditions, colleagues and supervisors. Specifically, cases might have reported systematically higher exposure levels, which may have resulted in an underestimation of risks.

The study may be facing a problem related to the choice of psychosocial exposures, despite the fact that our survey data are based on well established concepts and validated tools [1]. There is accumulating evidence that other conceptualisations of psychosocial work exposures, such as the effort-reward imbalance model [53] or the organisational justice model [54,55] may prove to be good predictors of mental health outcomes.

The use of the psychosocial JEM has limitations in assessing work settings and work organisation related variables. Future developments of models and measures of psychosocial work environment exposures are needed [56,42].

## Conclusion

Psychosocial work conditions such as job control, emotional demands and working with people, play a role in the risk of developing psychiatrically diagnosed depression and anxiety disorders but the risk pattern differs as between the sexes. Convincing explanation for these differences is yet to be found and more studies on gender dependent, work related risk factors are desirable. Additionally, the findings seem to indicate that there may be difference in determinants of hospitalisation and common mental health problems. There is scope for further research on the pathways between subclinical mental health problems, a diagnosed mental disorder and work related risk factors.

## Competing interests

The authors declare that they have no competing interests.

## Authors' contributions

JW participated in the study design, analysed and interpreted the data and took primary responsibility for writing the paper. JPB contributed to the study design, the interpretation of the findings and the preparation of the manuscript. EA participated in the study design and data collection, provided statistical advice and help in editing the manuscript. PBM contributed to the study design, results interpretation and revised the manuscript. HB and FT provided a part of the data, help in interpreting the data and revised the manuscript. All authors read and approved the final manuscript.

## Pre-publication history

The pre-publication history for this paper can be accessed here:



## References

[B1] Karasek R, Brisson C, Kawakami N, Houtman I, Bongers P, Amick B (1998). The Job Content Questionnaire (JCQ): an instrument for internationally comparative assessments of psychosocial job characteristics. J Occup Health Psychol.

[B2] Levi L, Bartley M, Marmot M, Karasek R, Theorell T, Siegrist J, Peter R, Belkic K, Savic C, Schnall P, Landsbergis P (2000). Stressors at the workplace: theoretical models. Occup Med.

[B3] D'Souza RM, Strazdins L, Lim LL, Broom DH, Rodgers B (2003). Work and health in a contemporary society: demands, control, and insecurity. J Epidemiol Community Health.

[B4] Mausner-Dorsch H, Eaton WW (2000). Psychosocial work environment and depression: epidemiologic assessment of the demand-control model. Am J Public Health.

[B5] de Lange AH, Taris TW, Kompier MA, Houtman IL, Bongers PM (2003). "The very best of the millennium": longitudinal research and the demand-control-(support) model. J Occup Health Psychol.

[B6] Stansfeld SA, Fuhrer R, Shipley MJ, Marmot MG (1999). Work characteristics predict psychiatric disorder: prospective results from the Whitehall II Study. Occup Environ Med.

[B7] de Jonge J, Bosma H, Peter R, Siegrist J (2000). Job strain, effort-reward imbalance and employee well-being: a large-scale cross-sectional study. Soc Sci Med.

[B8] Brotheridge CM, Grandey AA (2002). Emotional labor and burnout: Comparing two perspectives of "people work". Journal of Vocational Behavior.

[B9] Henderson A (2001). Emotional labor and nursing: an under-appreciated aspect of caring work. Nurs Inq.

[B10] Zerbe WJ, Ashkanasy NM, Härtel CEJ and Zerbe WJ (2000). Emotional dissonance and employee well-being. Emotions in the workplace: Research, theory and practice.

[B11] Andrea H, Bultmann U, Beurskens AJ, Swaen GM, van Schayck CP, Kant IJ (2004). Anxiety and depression in the working population using the HAD Scale--psychometrics, prevalence and relationships with psychosocial work characteristics. Soc Psychiatry Psychiatr Epidemiol.

[B12] Bultmann U, Kant IJ, Van den Brandt PA, Kasl SV (2002). Psychosocial work characteristics as risk factors for the onset of fatigue and psychological distress: prospective results from the Maastricht Cohort Study. Psychol Med.

[B13] Kivimaki M, Elovainio M, Vahtera J, Virtanen M, Stansfeld SA (2003). Association between organizational inequity and incidence of psychiatric disorders in female employees. Psychol Med.

[B14] Ylipaavalniemi J, Kivimaki M, Elovainio M, Virtanen M, Keltikangas-Jarvinen L, Vahtera J (2005). Psychosocial work characteristics and incidence of newly diagnosed depression: a prospective cohort study of three different models. Soc Sci Med.

[B15] Gerberich SG, Church TR, McGovern PM, Hansen HE, Nachreiner NM, Geisser MS, Ryan AD, Mongin SJ, Watt GD (2004). An epidemiological study of the magnitude and consequences of work related violence: the Minnesota Nurses' Study. Occup Environ Med.

[B16] Menckel E, Viitasara E (2002). Threats and violence in Swedish care and welfare--magnitude of the problem and impact on municipal personnel. Scand J Caring Sci.

[B17] Wieclaw J, Agerbo E, Mortensen PB, Burr H, Tüchsen F, Bonde JP (2006). Work-related violence and threats and the risk of depression and stress-related disorders. J Epidemiol Community Health.

[B18] Hogh A, Henriksson ME, Burr H (2005). A 5-year follow-up study of aggression at work and psychological health. Int J Behav Med.

[B19] der van Doef M, Maes S (1999). The Job Demand-Control(-Support) Model and psychological well-being: a review of 20 years of empirical research. Work & Stress.

[B20] Stansfeld S, Candy B (2006). Psychosocial work environment and mental health--a meta-analytic review. Scand J Work Environ Health.

[B21] Bonde JP (2008). Psychosocial factors at work and risk of depression: a systematic review of the epidemiological evidence. Occup Environ Med.

[B22] Kasl SV (1998). Measuring job stressors and studying the health impact of the work environment: an epidemiologic commentary. J Occup Health Psychol.

[B23] Teschke K, Olshan AF, Daniels JL, De Roos AJ, Parks CG, Schulz M, Vaughan TL (2002). Occupational exposure assessment in case-control studies: opportunities for improvement. Occup Environ Med.

[B24] Kauppinen T, Toikkanen J, Pukkala E (1998). From cross-tabulations to multipurpose exposure information systems: a new job-exposure matrix. Am J Ind Med.

[B25] Benke G, Sim M, Fritschi L, Aldred G, Forbes A, Kauppinen T (2001). Comparison of occupational exposure using three different methods: hygiene panel, job exposure matrix (JEM), and self reports. Appl Occup Environ Hyg.

[B26] Johnson JV, Stewart WF (1993). Measuring work organization exposure over the life course with a job-exposure matrix. Scand J Work Environ Health.

[B27] Cohidon C, Niedhammer I, Wild P, Gueguen A, Bonenfant S, Chouaniere D (2004). Exposure to job-stress factors in a national survey in France. Scand J Work Environ Health.

[B28] Amick BC, McDonough P, Chang H, Rogers WH, Pieper CF, Duncan G (2002). Relationship between all-cause mortality and cumulative working life course psychosocial and physical exposures in the United States labor market from 1968 to 1992. Psychosom Med.

[B29] Schwartz JE, Pieper CF, Karasek RA (1988). A procedure for linking psychosocial job characteristics data to health surveys. Am J Public Health.

[B30] Bultmann U, Kant IJ, Schroer CA, Kasl SV (2002). The relationship between psychosocial work characteristics and fatigue and psychological distress. Int Arch Occup Environ Health.

[B31] Johnson JV, Stewart W, Hall EM, Fredlund P, Theorell T (1996). Long-term psychosocial work environment and cardiovascular mortality among Swedish men. Am J Public Health.

[B32] Hammar N, Alfredsson L, Johnson JV (1998). Job strain, social support at work, and incidence of myocardial infarction. Occup Environ Med.

[B33] Andersen I, Burr H, Kristensen TS, Gamborg M, Osler M, Prescott E, Diderichsen F (2004). Do factors in the psychosocial work environment mediate the effect of socioeconomic position on the risk of myocardial infarction? Study from the Copenhagen Centre for Prospective Population Studies. Occup Environ Med.

[B34] Hemmingsson T, Lundberg I (1998). Work control, work demands, and work social support in relation to alcoholism among young men. Alcohol Clin Exp Res.

[B35] Seidler A, Nienhaus A, Bernhardt T, Kauppinen T, Elo AL, Frolich L (2004). Psychosocial work factors and dementia. Occup Environ Med.

[B36] Wieclaw J, Agerbo E, Mortensen PB, Bonde JP (2005). Occupational risk of affective and stress-related disorders in the Danish workforce.. Scand J Work Environ Health.

[B37] Borgan O, Langholz B (1993). Nonparametric estimation of relative mortality from nested case-control studies. Biometrics.

[B38] Kessing LV (1998). Validity of diagnoses and other clinical register data in patients with affective disorders. Eur Psychiatry.

[B39] Burr H, Bjorner JB, Kristensen TS, Tuchsen F, Bach E (2003). Trends in the Danish work environment in 1990-2000 and their associations with labor-force changes. Scand J Work Environ Health.

[B40] Michie S, Williams S (2003). Reducing work related psychological ill health and sickness absence: a systematic literature review. Occup Environ Med.

[B41] Tennant C (2001). Work-related stress and depressive disorders. J Psychosom Res.

[B42] Rugulies R, Bultmann U, Aust B, Burr H (2006). Psychosocial Work Environment and Incidence of Severe Depressive Symptoms: Prospective Findings from a 5-Year Follow-up of the Danish Work Environment Cohort Study. Am J Epidemiol.

[B43] Kristensen TS, Hannerz H, Hogh A, Borg V (2005). The Copenhagen Psychosocial Questionnaire--a tool for the assessment and improvement of the psychosocial work environment. Scand J Work Environ Health.

[B44] King G, Zeng L (2002). Estimating risk and rate levels, ratios and differences in case-control studies. Stat Med.

[B45] Paterniti S, Niedhammer I, Lang T, Consoli SM (2002). Psychosocial factors at work, personality traits and depressive symptoms. Longitudinal results from the GAZEL Study. Br J Psychiatry.

[B46] Niedhammer I, Goldberg M, Leclerc A, Bugel I, David S (1998). Psychosocial factors at work and subsequent depressive symptoms in the Gazel cohort. Scand J Work Environ Health.

[B47] Melchior M, Caspi A, Milne BJ, Danese A, Poulton R, Moffitt TE (2007). Work stress precipitates depression and anxiety in young, working women and men. Psychol Med.

[B48] Wieclaw J, Agerbo E, Mortensen PB, Bonde JP (2006). Risk of affective and stress related disorders among employees in human service professions. Occup Environ Med.

[B49] Ashkanasy NM, Härtel CEJ, Zerbe WJ, Ashkanasy NM and Härtel CE (2000). Emotions in the workplace: Research, theory, and practice. Emotions in the workplace: Research, theory, and practice.

[B50] Grandey AA (2000). Emotion regulation in the workplace: a new way to conceptualize emotional labor. J Occup Health Psychol.

[B51] van Vegchel N, Jonge J, Söderfeldt M, Dormann C, Schaufeli W (2004). Quantitative Versus Emotional Demands Among Swedish Human Service Employees: Moderating Effects of Job Control and Social Support. International Journal of Stress Management.

[B52] Johnson JV, Stewart WF (1993). Measuring work organization exposure over the life course with a job- exposure matrix. Scand J Work Environ Health.

[B53] van Vegchel N, de Jonge J, Bosma H, Schaufeli W (2005). Reviewing the effort-reward imbalance model: drawing up the balance of 45 empirical studies. Soc Sci Med.

[B54] Kivimaki M, Elovainio M, Vahtera J, Ferrie JE (2003). Organisational justice and health of employees: prospective cohort study. Occup Environ Med.

[B55] Elovainio M, Kivimaki M, Vahtera J (2002). Organizational justice: evidence of a new psychosocial predictor of health. Am J Public Health.

[B56] Kristensen TS, Bjorner JB, Christensen KB, Borg V (2004). The distinction between work pace and working hours in the measurement of quantitative demands at work. Work & Stress.

